# Selective Role of the Putamen in Serial Reversal Learning in the Marmoset

**DOI:** 10.1093/cercor/bhy276

**Published:** 2018-11-03

**Authors:** Stacey A W Jackson, Nicole K Horst, Sebastian F A Axelsson, Naotaka Horiguchi, Gemma J Cockcroft, Trevor W Robbins, Angela C Roberts

**Affiliations:** 1 Department of Psychology, University of Cambridge, Downing Street, Cambridge, UK; 2 Behavioural and Clinical Neuroscience Institute, University of Cambridge, Downing Street, Cambridge, UK; 3 Department of Physiology, Development and Neuroscience, University of Cambridge, Downing Street, Cambridge, UK

**Keywords:** caudate, cognitive flexibility, primate, rules, striatum

## Abstract

Fronto-striatal circuitry involving the orbitofrontal cortex has been identified as mediating successful reversal of stimulus-outcome contingencies. The region of the striatum that most contributes to reversal learning remains unclear, with studies in primates implicating both caudate nucleus and putamen. We trained four marmosets on a touchscreen-based serial reversal task and implanted each with cannulae targeting both putamen and caudate bilaterally. This allowed reversible inactivation of the two areas within the same monkeys, but across separate sessions, to directly investigate their respective contributions to reversal performance. Behavioral sensitivity to the GABA_A_ agonist muscimol varied across subjects and between brain regions, so each marmoset received a range of doses. Intermediate doses of intra-putamen muscimol selectively impaired reversal performance, leaving the baseline discrimination phase unchanged. There was no effect of low doses and high doses were generally disruptive. By contrast, low doses of intra-caudate muscimol improved reversal performance, while high doses impaired both reversal and baseline discrimination performance. These data provide evidence for a specific role of the putamen in serial reversal learning, which may reflect the more habitual nature of repeated reversals using the same stimulus pair.

## Introduction

Cognitive flexibility, defined as the ability of an organism to adapt its thinking or behavior to changing circumstances (Eslinger and Grattan 1993), is a key part of efficient executive function (Dajani and Uddin 2015). Greater cognitive flexibility is generally advantageous, particularly in dynamic environments, and in humans has been linked to superior functioning across multiple domains, including social ability (Bonino and Cattelino 1999) and emotion regulation (Hendricks and Buchanan 2016). Conversely, reduced cognitive flexibility is associated with various neuropsychiatric disorders including Obsessive Compulsive Disorder (OCD; Remijnse et al. 2005), addiction (Ersche et al. 2008; Izquierdo and Jentsch 2012), depression (Murphy et al. 2003; Robinson et al. 2012), and schizophrenia (Floresco et al. 2009; Leeson et al. 2009), as well as neurodevelopmental disorders such as autism spectrum disorder (Cruz et al. 2013).

Such is the importance of cognitive flexibility that it has been the subject of intensive research over the years, using a range of different paradigms (Kehagia et al. 2010; Cools 2015). Reversal learning is one such paradigm that has been well-validated (Izquierdo and Jentsch 2012). In a typical protocol, subjects must learn to discriminate between one of two stimuli to gain reward, only for the stimulus/action-outcome associations to then be reversed. The relative simplicity of the task has meant it has been possible to adapt the procedure to accommodate performance in a wide variety of species (Izquierdo et al. 2016), thus allowing considerable translational potential (Oikonomidis et al. 2016; Robbins 2017).

The orbitofrontal cortex (OFC) is widely accepted to be critical for reversal learning in rodents (Hamilton and Brigman 2015) and marmosets (Dias et al. 1996; Clarke et al. 2008; Oikonomidis et al. 2016). Although a specific role of the macaque OFC in reversal learning has been refuted (Rudebeck et al. 2013), a more posterolateral region has since been implicated (Chau et al. 2015). The striatum, which receives strong projections from the OFC (Haber et al. 1995; Roberts et al. 2007; Schilman et al. 2008; Heilbronner et al. 2016), also plays a major role. Thus, lesions or inactivations of the caudate nucleus (Divac et al. 1967) and nucleus accumbens (Stern and Passingham 1995) in rhesus monkeys and marmosets (Clarke et al. 2008), and the dorsomedial (Kirkby 1969; Ragozzino et al. 2002; Castañé et al. 2010) and ventral striatum (Annett et al. 1989; Ferry et al. 2000, but see Castañé et al. 2010) of the rat, have all been shown to induce reversal learning deficits. In addition, dopaminergic depletion within the medial caudate impairs reversal learning in marmosets (Clarke et al. 2011) as does a D2/3 receptor agonist infused into rat nucleus accumbens (Haluk and Floresco 2009).

Despite this consensus for a role of the OFC and medial/ventral striatum in reversal learning, a recent correlative study has advanced an alternative perspective implicating dopamine-dependent processes of the putamen interacting with serotonin-dependent OFC substrates in vervet monkeys (Groman et al. 2013). This combination of OFC serotonin and striatal dopamine, although broadly consistent with neurochemical findings in marmosets (Clarke et al. 2004, 2005, 2007, 2011), differs by virtue of it implicating the putamen rather than the caudate as the critical striatal locus.

Since a causal role for the putamen has been little studied with respect to reversal learning, the aim of the present investigation was to clarify the respective contributions of the primate anterior putamen and medial caudate. Marmosets were trained on a serial reversal learning paradigm (Rygula et al. 2010), the design of which supported the use of repeated, acute manipulations. The task comprised a daily baseline discrimination phase in which responding to one of two highly familiar stimuli was paired with reward and responding to the other produced negative feedback. This was followed by a phase in which the action-outcome contingencies were reversed. These reversed contingencies then formed the basis of the baseline discrimination phase on the next day, immediately followed by another reversal. The two phases within a daily session made it possible to isolate impairments specific to reversal learning from more general deficits affecting discrimination performance in both phases of the task. Marmosets were implanted with indwelling cerebral cannulae targeting the medial caudate and anterior putamen (as defined in Groman et al. 2013). These regions were then independently and reversibly inactivated using an individually tailored range of doses of the GABA_A_ agonist, muscimol.

## Materials and Methods

### Subjects and Housing

Four experimentally naïve marmosets (*Callithrix jacchus*; all male), bred on site at the University of Cambridge Marmoset Breeding Colony, were housed in pairs (male-female or male-male, males being vasectomized) in custom-made housing. Rooms were maintained at 22 ± 1 °C and 50 ± 1% relative humidity and were gradually illuminated from 7:00 to 7:30 am and dimmed from 7:00 to 7:30 pm, following a 12-h light/dark cycle with dawn and dusk. Marmosets received a nutritionally complete diet, which consisted of sandwiches, fruit, and rusk at the weekend, and a restricted but calorically equivalent diet of pellets and fruits or vegetables during the week. From Monday to Friday, access to water was restricted for 22 h out of every 24, with *ad libitum* access for the remaining two hours after behavioral testing, and *ad libitum* access over the weekend. The housing contained a range of environmental enrichment aids including ropes and rope ladders, and marmosets were given occasional treats after testing. Marmosets were weighed on a weekly basis and their welfare monitored by members of the research and husbandry teams. All procedures were carried out in accordance with the UK Animals (Scientific Procedures) Act 1986 as amended in 2012, under project licenses 80/2225 and 70/7618. In addition, the University of Cambridge Animal Welfare and Ethical Review Board provided ethical approval of the project license and its amendments, as well as individual studies and procedures via delegation of authorization to the Named Animal Care and Welfare Officer (NACWO) for individual study plans.

### Behavioral Testing Apparatus

**Figure 1.**
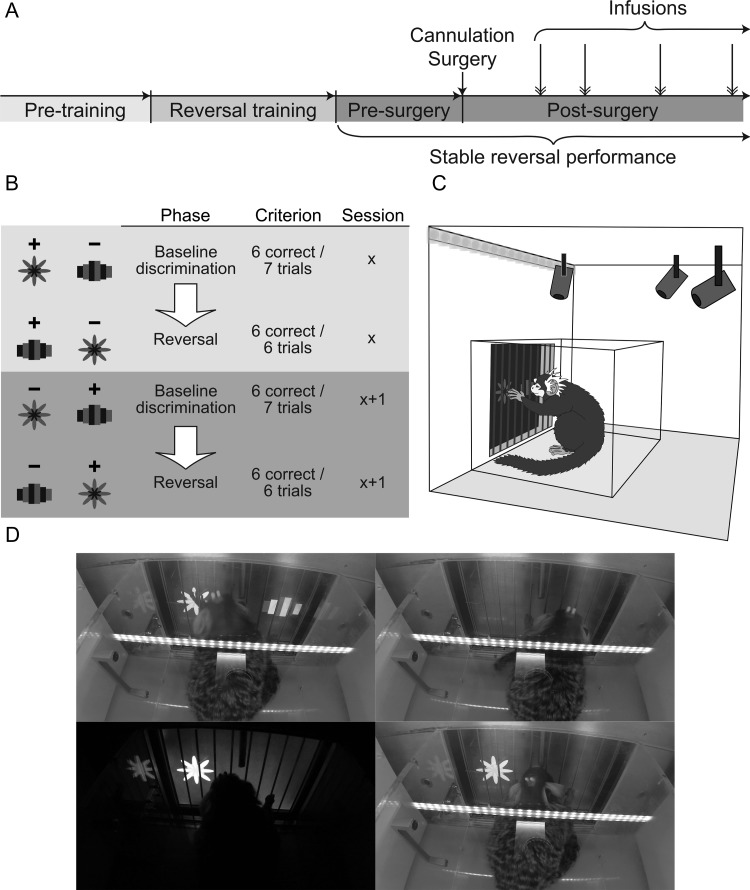
Schematic of task and experimental design. A. Timeline of experimental protocol. Naïve marmosets were taught to respond on the touchscreen “Pre-training”, and then to perform the serial reversal learning task “Reversal training”. Once marmosets were exhibiting stable reversal performance they underwent cannulation of the medial caudate and anterior putamen. Post-surgery, marmosets received intra-striatal infusions of the GABA_A_ agonist muscimol or saline control infusions on test days. B. Schema illustrating stimulus-outcome contingencies across two consecutive days. After reaching a behavioral criterion of six correct responses within seven trials in the baseline discrimination phase of a session, the stimulus-outcome contingencies were reversed, and the marmoset then had to achieve six correct responses within six trials to pass the reversal phase of the session. The next day, the stimulus-outcome contingency of the baseline discrimination phase was the same as that in the reversal phase of the previous day. C. Diagram illustrating the position of the marmosets within the behavioral testing apparatus and carrying box in relation to the touchscreen, houselights and video cameras. D. Photographs of a marmoset performing the serial reversal learning task, showing counter-clockwise from top left: the marmoset touching a stimulus, punishment darkness following an incorrect response, collection of banana-flavored milk reward following a correct response and an inter-trial interval.

Marmosets were tested once daily on Monday to Friday and given time off at the weekend. All marmosets were first trained to enter a Perspex carrying box in which they were transported to a behavioral testing apparatus within a darkened room. The apparatus was comprised of a custom-made sound-attenuated box containing a touch-sensitive computer monitor (“touchscreen”; NEX121 TFT LCD Monitor; Nexio). The carrying box was placed within the apparatus, with one side removed to enable access to the touchscreen through a vertical array of metal bars. A centrally placed licking spout allowed the delivery of cooled banana-flavored milk (made with Nesquik powder; Nestlé) as the positive reinforcer. Licking was detected by the interruption of an infrared photobeam situated at the mouth of the licking spout. A speaker at the back of the chamber played the sounds used in the experiments: a birdsong recording, which acted as a cue to collect reward, or a ~100 dB brief (0.3 s) auditory stimulus used to signal incorrect choices. Stimulus presentation upon the touchscreen, the speaker, and the reinforcer pumps was controlled by modules within the MonkeyCantab program (v9.3–11.2, R.N. Cardinal) developed from MonkeyCantab (Weed et al. 1999; originally designed by Robbins and Roberts) and using the Whisker control system (Cardinal and Aitken 2010) via an operant chamber interface (Biotronix). All experiments were monitored in real-time by video cameras mounted to the roof of the touchscreen chamber. A schematic of the testing apparatus and still images from a behaving animal recorded from a mounted video camera are shown in Figures 1C–D.

### Preliminary Behavioral Training

Marmosets were acclimatized to the carrying box, touchscreen chamber, and banana-flavored milk reinforcer. They were then gradually trained, in a manner first described in Roberts et al. (1988), to respond on the touchscreen for reward. Marmosets were then moved to a “lick contingent” condition where, upon touching a stimulus presented on the touchscreen, “birdsong” would be played to cue reward availability, and reward delivery would begin when the marmoset began to lick at the licking spout, as detected by the interruption of the infrared photobeam. All sessions were twenty minutes long.

### Training on Serial Reversal Learning Paradigm

Subjects were given two training visual discriminations in the “Pre-training” phase and then moved to the main discriminative set (Fig. 1) for initial between-session reversal training. Reward delivery and inter-trial interval (ITI) remained the same as in the final stages of preliminary behavioral training, with 5 s of banana-flavored milk delivered lick-contingently with a “birdsong” cue upon response to the correct stimulus, and an auditory cue (0.3 s long at a volume of ~100 dB) in addition to 5 s of darkness when marmosets made an incorrect choice. The selected visual stimulus remained on the screen during the 5 s of either reward delivery or darkness. The daily testing session terminated after a subject reached the criterion of six consecutive correct responses, or failing that, after 20 min had elapsed. Upon reaching criterion, stimulus-reward contingencies were reversed in the subsequent session, such that the previously correct stimulus became incorrect and the previously incorrect stimulus became correct. Marmosets were tested on the new stimulus-reward contingencies until they re-gained criterion. The response-outcome contingencies were again reversed for the subsequent session, and marmosets continued to be tested on these “between-session” reversals until they could consistently, upon receiving reversed response-outcome contingencies, learn the reversed contingencies within the session—a “same-day pass”. Marmosets progressed to “within-session” reversals when they had achieved 10 same-day passes, though these did not need to be consecutive.

### Within-Session Serial Reversal Learning Paradigm

Within-session reversals comprised a baseline discrimination phase where response-outcome contingencies were the same as those at the end of the previous day, and a reversal phase, where they were inverted. Upon six correct responses within seven consecutive trials (baseline discrimination phase), the response-outcome contingencies were reversed (reversal phase). There were no environmental signals that cued the transition between phases other than the change in response-outcome contingencies. The testing session terminated after either marmosets reached the criterion of six consecutive correct responses on the reversal phase, or failing that, 20 min had elapsed. In the event that a subject did not pass the baseline discrimination phase of a session, the response-outcome contingencies at the beginning of the baseline discrimination phase of the next session would remain the same as those of the previous day’s failed baseline discrimination phase. If a subject did not pass the reversal phase of the session, the response-outcome contingencies of the following day’s baseline discrimination phase were the same as those in the failed reversal phase. Once a marmoset had exhibited stable reversal performance, as defined by the successful completion of ten within-session reversals, they underwent cannulation surgery and, after recovery, received intra-striatal infusions of saline vehicle or the GABA_A_ agonist muscimol to inactivate the anterior putamen or medial caudate on test serial reversal sessions interspersed between non-infusion sessions.

### Cannulation Surgery

Marmosets were pre-medicated using 0.1 mL (100 mg/mL) of the anesthetic ketamine hydrochloride i.m. (Vetelar; Amersham Biosciences and Upjohn) and 0.03 mL of 50 mg/mL of the analgesic carprofen s.c. (Carprieve; Pfizer). They were intubated and anesthesia maintained by administration of 2.0%–2.5% isoflurane in 0.3 L/min O_2_, and then placed upon a heat mat positioned within a stereotaxic frame modified for the marmoset (David Kopf). Pulse rate, O_2_ saturation, breathing rate, and CO_2_ saturation were all monitored by pulse oximetry and capnography (Microcap Handheld Capnograph; Oridion Capnography), and core body temperature was monitored by a rectal thermometer (MicroTherma 2 T digital thermometer; ThermoWorks). The percentage of isoflurane in the isoflurane/O_2_ mixture and the heat mat temperature were modulated during surgery in response to changes in vital signs, 1.0 mL of warmed saline was given s.c. every 90 min to prevent dehydration, and the hind legs and body were turned every hour to stimulate blood flow.

Indwelling guide cannulae (Plastics One) were implanted to target the ventromedial caudate and putamen; the double guide cannulae (26-gauge, 2.4 mm c/c) were custom-made so that one guide targeted the ventromedial caudate (8 mm in length) and the other, the putamen (9 mm in length) at coordinates of anteroposterior (AP) + 11, lateromedial (LM) ± 2.55, ventral (V) + 11.5 and AP + 11, LM ± 4.95, V + 10.5, respectively. AP Coordinates were adjusted where necessary *in situ* according to a prefrontal cortical depth procedure described in Roberts et al. (2007). Cannulae were fixed in place with the aid of an array of steel skull screws (Plastics One) and the application of an adhesive (Super-Bond C&B; Sun Medical Co.) across the skull surface, as well as the application of dental acrylic (Paladur, Kulzer, Mitsui Chemicals Group) to the surrounds of the guide cannulae. Wire stylets were inserted to occlude the cannulae and protective caps screwed on top. Postoperatively, and when fully recovered (usually within 3-4 h) all monkeys were returned to their home cage and then received 0.1 mL of the analgesic agent meloxicam (1.5 mg/mL oral suspension; Boehringer Ingelheim) for 3 days, after which they received a further “rest” period (weekend food, ad libitum water, and no behavioral testing) of at least 1 week. Cannulae were cleaned every week (and caps and cannula occluders changed) to ensure the cannulae remained patent and the implant site free from infection.

### Post-Mortem Assessment of Cannula Placement

**Figure 2.**
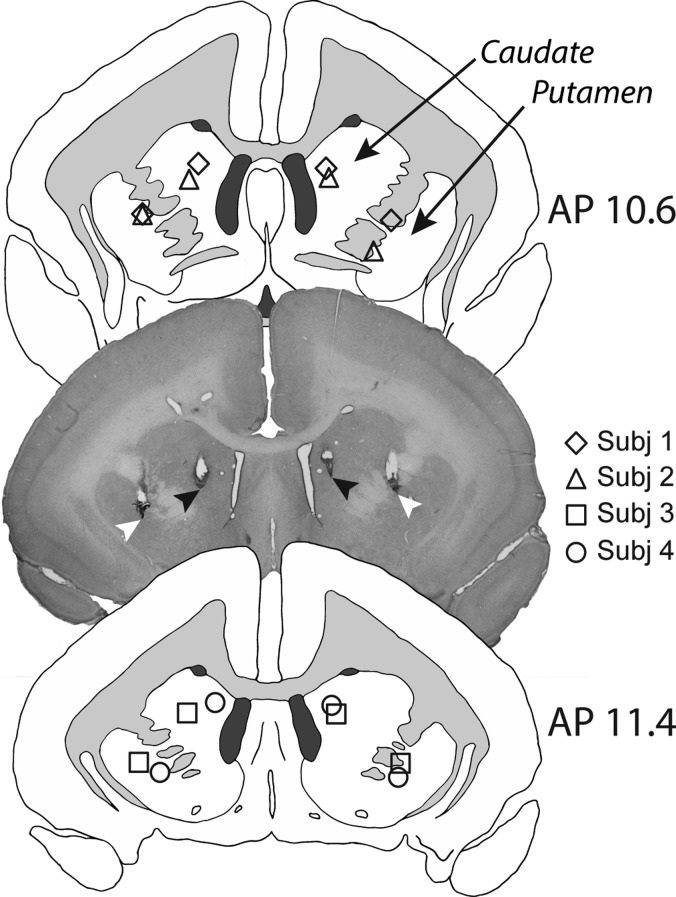
Schematics showing the intra-cerebral cannulae placements in the medial caudate and putamen for each subject, in AP planes 10.6 and 11.4. In the representative histological section (taken from Subject 3), black arrows show the placement of the tip of the infusion cannulae in the medial caudate, and white arrows the placement of the tip of the infusion cannulae in the anterior putamen.

Animals were pre-medicated with 0.1 mL i.m. of ketamine hydrochloride (Vetalar; 0.05 mL of a 100-mg solution, i.m.; Amersham Biosciences and Upjohn) before being euthanized with 1.0 mL i.v. of pentobarbital sodium (Dolethal; 200 mg/mL solution; Merial Animal Health). Animals were then perfused transcardially with 500 mL 0.1 M PBS solution, followed by 500 mL of 4% paraformaldehyde fixative solution. The brain was removed and left in the 4% paraformaldehyde fixative solution overnight before being transferred to 30% sucrose–PBS solution for at least 48 h. Brains were then sectioned on a freezing microtome (coronal sections; 60 μm), mounted on gelatin-subbed slides, and stained with cresyl fast violet. The sections were viewed under a Leitz DMRD microscope (Leica Microsystems). The cannula locations for each animal were schematized onto drawings of standard marmoset brain coronal sections, and composite diagrams were then made to illustrate the extent of overlap between animals (Fig. 2). A histological section is also presented to illustrate cannulae placements in both the medial caudate and anterior putamen in an individual subject.

### Intra-cerebral Drug Infusion Protocol

Intra-cerebral drug infusions were conducted according to standardized laboratory protocols as previously described (Clarke et al. 2015), under sterile conditions. The marmoset was gently restrained by an assistant, the caps and cannula occluders were removed from the guide cannulae, and the site was cleaned with 70% isopropyl alcohol. An injector (Plastics One) connected to a 10 μL Hamilton syringe in a syringe pump was inserted into the guide cannula so as to protrude at least 0.5 mm from the tip of the guide, and 0.5 μL of saline or 0.03–0.3 μg muscimol in 0.5 μL saline was infused bilaterally into either medial caudate or putamen at a rate of 0.25 μL/min. The consensus from previous studies using fluorescent-tagged (Allen et al. 2008) or radio-labeled (Sperber et al. 1989; Krupa and Thompson 1997; Martin and Ghez 1999) muscimol and/or glucose metabolism (Martin and Ghez 1999) is an effective radius of ~1.5 mm for the inactivating effects of muscimol at doses, volumes, and infusion rates similar to those used here. Thus, with injector tips located 2.6 mm apart, we do not expect infusions in one striatal region to affect the other area directly. Following the infusion, the injector was left in place for a further minute to allow the drug to diffuse before injector removal. Fresh, sterile occluders and caps were replaced, and the marmoset was returned to its home cage for 40 min before the behavioral testing session commenced. The number of infusions received per animal (putamen, caudate) was as follows: Subject 1 (2, 2); Subject 2 (4, 2); Subject 3 (6, 3); Subject 4 (4, 3).

### Determination of Muscimol Dose Range and Intra-cerebral Infusion Regimen

Marmosets completed a sequence of muscimol infusions into the anterior putamen before receiving a similar sequence into the medial caudate nucleus. Individuals showed differential behavioral sensitivity to specific doses of intra-striatal muscimol, but common patterns of ordinal dose-dependent effects were apparent after muscimol infusions in both putamen and caudate. We adopted a stringent procedure for determining the individual dose response ranges (see Table 1). All animals initially received an infusion of 0.3 μg into the putamen. A lower dose of 0.1 μg was subsequently administered either when failure to respond resulted in premature disengagement from the task (thus, precluding accurate measures of reversal learning performance) or if an animal exhibited generalized deficits across both task phases. Depending on the response to this dose, further doses were administered either above (0.18 μg) or below 0.1 μg (0.03 and 0.06 μg) in order to define the graded nature and selectivity of any dose-related behavioral effects. Our primary interest was to determine the dose at which each animal showed behavioral effects in the reversal phase only.

**Table 1.** Doses of muscimol infused into the putamen. All animals initially received an infusion of 0.3 μg into the putamen (dose denoted by †). Animals that were grossly impaired by this dose were then given lower doses in an iterative fashion to determine the dose that produced a selective reversal impairment on the error difference score, as compared to saline. Dosing categories are denoted by labels as follows: Low_p_ = putamen low, Int_p_ = putamen intermediate, High_p_ = putamen high, H+_p_ = a high putamen dose that produced general impairment in Subject 2. Gray shading reflects a significant impairment. A superscript “F” indicates failure to reach behavioral criterion. The intermediate doses from putamen infusions were used as the first dose in caudate infusions.

	Baseline discrimination					Reversal					
*Subject*	*0.03 μg*	*0.06 μg*	*0.1 μg*	*0.18 μg*	*0.3 μg*^†^	*0.03 μg*	*0.06 μg*	*0.1 μg*	*0.18 μg*	*0.3 μg*^†^
1					Int_p_					Int_p_
2	Low_p_	Int_p_	High_p_		**H+**_**p**_^**F**^	Low_p_	Int_p_	**High**_**p**_^**F**^		
3		Low_p_	Int_p_		High_p_		Low_p_	Int_p_^F^		**High**_**p**_^**F**^
4			Low_p_	Int_p_	High_p_			Low_p_	**Int**_**p**_^**F**^	**High**_**p**_^**F**^

For each subject the highest dose that produced a selective behavioral effect on reversal in the putamen was then chosen to be the first dose infused into the medial caudate nucleus (see Table 2). In cases where the initial dose produced either no impairment, premature response disengagement, or generalized disruption of performance across both task phases, a higher or lower dose was subsequently infused accordingly. Whereas the dose-dependent effects of intra-putamen muscimol were described by three doses (low, intermediate, and high), in caudate, animals displayed only two distinct effects; thus the dosing categories for caudate infusions are only low and high. Note that the low and high doses for caudate are not necessarily the same absolute doses as those producing low and high-dose effects in the putamen. The relationships between doses are described in Tables 1 and 2. The first animal to receive a putamen infusion (Subject 1) showed the selective reversal effect with the initial 0.3 μg dose, which was then infused into the caudate. Unfortunately, the implant of this animal was then irreparably damaged, and we were unable to establish any further dose-dependent effects in either area.

**Table 2.** Doses of muscimol infused into the caudate. All animals initially received an infusion of the muscimol dose that produced a selective reversal effect in the putamen (dose denoted by underline). This initial dose produced either a selective reversal improvement (superscript “I”, Subject 1) or impairment (gray shading, Subjects 2–4) across both task phases, as measured against saline performance. Whilst subjects varied in their behavioral sensitivity to specific doses of muscimol, overall, lower caudate doses (Low_c_) produced a selective reversal improvement whilst higher caudate doses (High_c_) caused baseline discrimination impairments. A superscript “F” indicates failure to reach behavioral criterion. Subject 1 received only a single dose, due to implant damage sustained after the first caudate infusion.

	Baseline discrimination					Reversal				
*Subject*	*0.03 μg*	*0.06 μg*	*0.1 μg*	*0.18 μg*	*0.3 μg*	*0.03 μg*	*0.06 μg*	*0.1 μg*	*0.18 μg*	*0.3 μg*
1					Low_c_					Low_c_^**I**^
2		High_c_					High_c_			
3		Low_c_	High_c_				Low_c_^I^	High_c_		
4			Low_c_	High_c_				Low_c_^I^	**High**_**c**_^**F**^	

Control saline infusions were interpolated randomly within the infusion series for the putamen and caudate nucleus. Marmosets received one or two infusions per week depending upon the stability of their performance. Infusions were administered at least 48 h apart from one another.

### Behavioral Measures

The main behavioral measures were the numbers of errors and trials in the baseline discrimination and reversal phases of the task on the infusion session and the preceding control session. To better understand the nature of any differences in post-infusion performance we also assessed “strategy” in terms of response types following rewarded vs. unrewarded trials (e.g., win-stay/lose-shift) and plotted errors against trials to produce learning curves for the reversal phase. Latencies were assessed to determine whether intra-striatal muscimol affected motor responses following rewarded and unrewarded responses.

### Statistical Approach and Data Analysis

Intra-striatal muscimol inactivation data were analyzed using mixed-model analyses of variance (ANOVAs), which were programmed using the statistical computing language R, version 3.3.1 with the Mac GUI R.app version 1.68. Linear mixed-effects modeling was achieved with the lme4 package (Bates et al. 2014), with statistical tests applied with the lmerTest package (Kuznetsova et al. 2016) using Type III sums of squares with the Satterthwaite approximation for degrees of freedom. Interactions and other effects were further investigated using general linear hypothesis tests (glht in multcomp package; Hothorn 2017) on the estimated marginal means with the Holm adjustment for multiple comparisons.

For the purposes of statistical analysis we computed difference scores for errors made or trials completed on the infusion day minus the immediately preceding control session when no infusion was given (non-infusion day). There was marked individual variation in normal non-infusion performance, and the difference score reduced the contribution of this variation to the model by allowing each animal to act as its own control. For the independent variable of muscimol dose in the putamen, we defined three levels, low, intermediate and high. The high dose was that producing either generalized behavioral disruption involving impairment in baseline discrimination as well as reversal or premature task disengagement during the reversal. The intermediate dose was the highest dose producing a selective reversal effect, the low dose having weaker or no effect. A similar categorization of doses was applied to the caudate data, with high-dose intra-caudate muscimol producing a generalized deficit across both task phases or premature response disengagement and low dose producing no impairments. There was no intermediate dose effect in the caudate.

ANOVA was performed for the putamen and caudate inactivations separately. Fixed factors chosen for the initial ANOVA included the session phase, i.e., baseline discrimination or reversal (“Phase”), and the dose of muscimol and saline (“Dose”), while subject was modeled as a random factor. The high-dose was not included in the analysis for the putamen because of the premature task disengagement in some animals, which meant that error scores were artificially low. Post hoc analyses were performed using separate independent mixed-model ANOVAs on the baseline discrimination and reversal phases. Further pairwise comparisons were made between individual data points based on the estimated marginal means with the Bonferroni–Holm adjustment.

Given the previously demonstrated perseverative nature of the reversal impairment following lesions of the caudate nucleus in marmosets (Clarke et al. 2008), muscimol-related selective reversal impairments were assessed for evidence of perseveration. This was achieved using two methods. First, counts of errors committed prior to the first correct post-reversal response were compared between the infusion day and the preceding non-infusion control session for impairing muscimol doses and saline. A mixed-model ANOVA of these data was followed by *post hoc* analysis of any effects by general linear hypothesis testing. Because some animals committed correct responses spuriously even in the first reversal trial, we additionally used signal detection theory to classify whether responding on the previously correct stimulus during the first six trials after reversal was above, at, or below chance (see Clarke et al. 2004, 2005 for details of this analysis method). We chose to only analyze the first six trials, as this was the minimum number of trials required to pass the reversal criterion and all animals completed at least this many trials if they reached the reversal phase. We then used Fisher’s Exact Test to assess whether the proportion of animals that showed a perseverative response profile after muscimol infusion-induced impairment was higher than was observed after saline infusions.

Impairments and improvements were further investigated to determine whether changes in performance following muscimol infusions were due to changes in responding after rewarding or non-rewarding feedback. The probability of a “win-stay” response (P(Stay | Win)) is calculated by dividing the number of times an animal selects the correct stimulus after having just received a reward for responding on the same stimulus by the total number of correct responses. The probability of a “lose-shift” (P(Shift | Loss)) response is determined by dividing the number of times the alternative stimulus is selected after an error by the total number of errors. The optimum strategy is to maximize both win-stay and lose-shift responses. The impact of intra-striatal muscimol on strategy usage was examined using separate repeated measures (rm)ANOVAs (aov_car from afex package; Singmann et al. 2018) of win-stay and lose-shift probabilities with the within-subject factor of Day (infusion day vs. preceding non-infusion day).

Median response latencies were calculated for correct and incorrect responses and a difference score taken between infusion sessions and non-infusion control sessions. A three-way mixed-model ANOVA with task phase, response outcome, and dose was followed by post hoc analyses, as described above.

## Results

### Cannulation Placements

The tips of the infusion cannulae could be visualized in the histological sections of each animal as illustrated in the photomicrograph of an exemplar animal in Figure 2. In all cases, the placements were located in the medial caudate and anterior putamen with a maximum 0.8 mm variation in the location of putamen placements between animals and the same variation in location of caudate placements between animals (see schematics in Fig. 2). There was little visible sign of any non-specific tissue damage around the cannula tips (see representative section in Fig. 2).

### Performance on the Serial Reversal Task Remained Stable Across Sessions

To determine whether implantation of cannulae or repeated infusions affected behavioral performance, we used rmANOVAs to compare average performance (mean of four sessions, two of each stimulus-outcome configuration) at three time points: prior to cannula implantation, after cannula implantation but before the first infusion, and at the end of the study. There was no effect of time point on either errors (*F*(2,6) = 1.15, *P* = 0.38) or trials (*F*(2,6) = 1.81, *P* = 0.24), nor were there interactions between time point and task phase (errors: *F*<1; trials: *F*<1). Only task phase significantly impacted performance in any way (errors: *F*(1,3) = 35.1, *P* = 0.0096; trials: *F*(1,3) = 29.4, *P* = 0.012).

Similarly, since all animals experienced intra-putamen infusions prior to intra-caudate infusions, we compared average performance on control sessions preceding infusions within each striatal region to determine whether variation in performance over time could account for any differences in the behavioral responses to muscimol between striatal regions. There were no striatal region-related differences in the number of errors on preceding sessions for either the reversal phase (paired *t*-tests of putamen vs. caudate; putamen = 17.25 ± 2.50 errors (44.4 ± 5.54 trials); caudate = 14.88 ± 2.14 errors (38.7 ± 4.51 trials); *F*<1 (*F*<1)) or for the baseline discrimination (putamen = 3.69 ± 1.12, errors (11.6 ± 2.16 trials); caudate = 6.21 ± 0.50 errors (17.6 ± 1.12 trials); *F*<1 (*F*<1)).

### Intra-Putamen Muscimol Infusions Produced Dose-Dependent Selective Impairments in Reversal Learning

**Figure 3.**
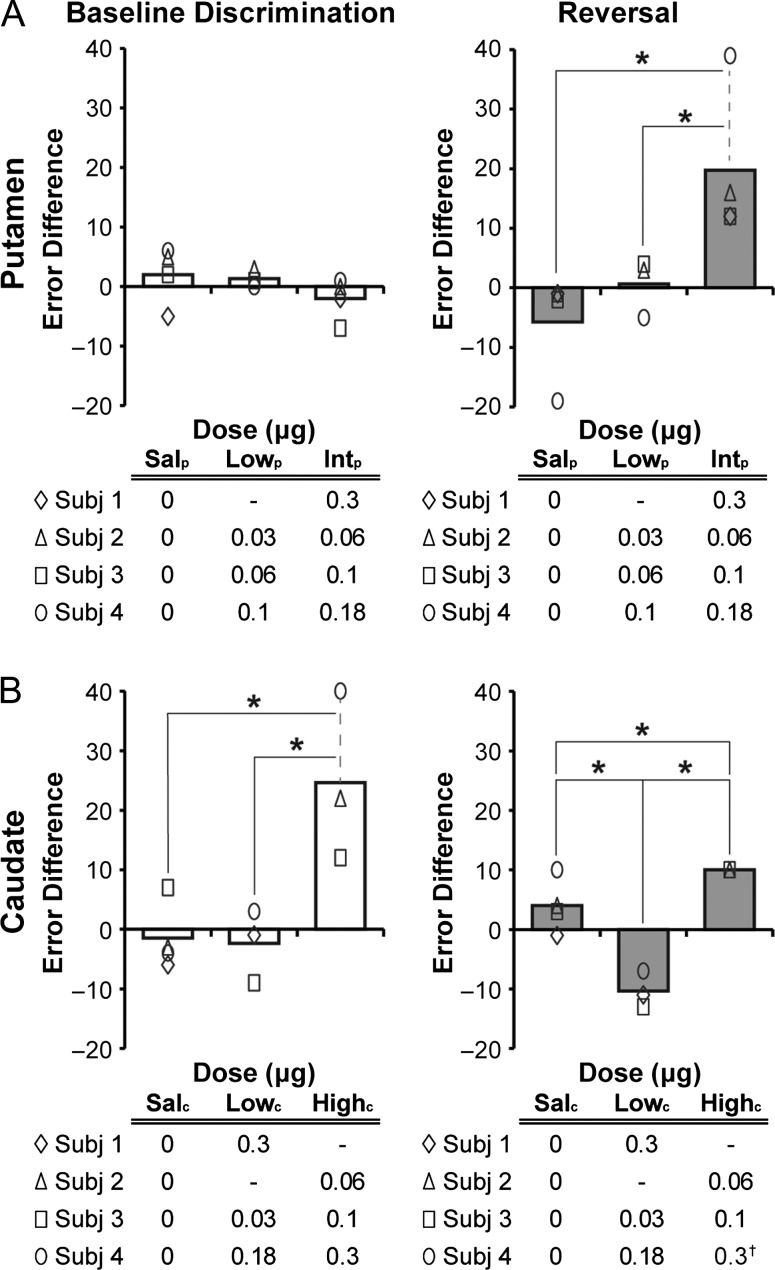
Effects of intra-putamen (A) and intra-caudate (B) administration of muscimol and saline on the error difference score (number of errors in the infusion session minus errors from the preceding control session) in the baseline discrimination and reversal phases of the task. The bars in each plot represent the mean for saline and varying doses of intra-striatal muscimol (Sal = saline, Low = lower dose, Int = intermediate dose, High = higher dose, with subscripts “p” for “putamen” and “c” for “caudate”). Data for individual subjects are denoted by the symbols defined in the tables below each plot, where specific doses are also tabulated. †indicates that Subject 4’s high-dose data was not included in the analysis of reversal performance (see Results). * = *P* < 0.05 in post hoc tests.

Intra-putamen muscimol infusions induced graded, dose-dependent deficits across the two phases of the task, the range of doses inducing these graded effects varying across individuals (Fig. 3A). Although behavioral sensitivity to specific doses varied between marmosets, the consistent ordinal pattern of effects allowed us to classify doses as low, intermediate, or high. Neither the low nor the intermediate dose had any effect on the baseline discrimination phase. However, all four animals exhibited selective deficits at the reversal phase following intermediate doses. At high doses, all animals displayed a generalized deficit across baseline discrimination and reversal phases or stopped responding during the reversal phase.

A mixed-model ANOVA confirmed a selective effect on errors in reversal following intermediate doses, as shown by a significant Phase by Dose interaction (*F*(2,16) = 8.26; *P* = 0.00343). Separate analysis of the two phases revealed that intra-putamen muscimol did not affect the baseline discrimination phase (*F*(2,4.95) = 1.50; *P* = 0.311), but did produce a significant deficit in the reversal phase (*F*(2,8) = 7.04; *P* = 0.0172). Pairwise comparisons of muscimol doses with saline showed that only intermediate doses produced significantly more errors (Intermediate vs. Saline: *P* < 0.001, Intermediate vs. Low: *P* < 0.05, Low vs. Saline *P* > 0.05). There were no differences in performance across non-infusion sessions as measured by a raw error count (Supplementary Fig. S1A). A mixed-model ANOVA confirmed an effect of reversal (*F*(1,13.3) = 75.2, *P* = 7.7 × 10^−7^), but no effect of Dose (*F*<1) or Phase × Dose interaction (*F*(2,13.3) = 1.99, *P* = 0.17).

The number of trials completed was not as strongly influenced by intra-putamen muscimol infusions (Supplementary Fig. S2A). There was a trend toward significant interaction between Phase and Dose (*F*(2,16) = 3.29, *P* = 0.064) on number of trials, which was driven by a trend toward an increase in trials at the intermediate muscimol dose (Intermediate vs. Saline: *P* = 0.058, Intermediate vs. Low: *P* > 0.05, Low vs. Saline *P* > 0.05) in the reversal phase.

There were no effects of intermediate- or high-dose intra-putamen muscimol on the number of errors preceding the first post-reversal correct response (Day: *F*<1, Dose: *F*(2,13.9) = 1.37, *P* = 0.29; Day × Dose: *F*(2,13.9) = 0.48, *P* = 0.63). The analysis based on signal detection theory classified three of four animals as perseverative in the first six reversal trials after the intermediate muscimol dose, with no animals showing perseveration in the preceding non-infusion sessions. The serial nature of this task reduces the number of perseverative errors committed upon reversal considerably, compared to non-serial reversal learning tasks when animals are less familiar with the concept of reversing (e.g., Clarke et al. 2004, 2008).

Analysis of response strategy following positive and negative feedback was conducted to gain further insight into the nature of the intermediate dose muscimol impairment (Supplementary Fig. S3). There was a significant decrease in the probability of sticking to the previously selected stimulus after receiving reward (“win-stay”) after intermediate intra-putamen muscimol as compared to non-infusion performance (*F*(1,3) = 147.3, *P* = 0.0012). A similar decrease in shifting away from the stimulus now associated with negative feedback (“lose-shift”) was present at a trend level after intermediate muscimol doses (*F*(1,3) = 6.66, *P* = 0.082).

**Figure 4.**
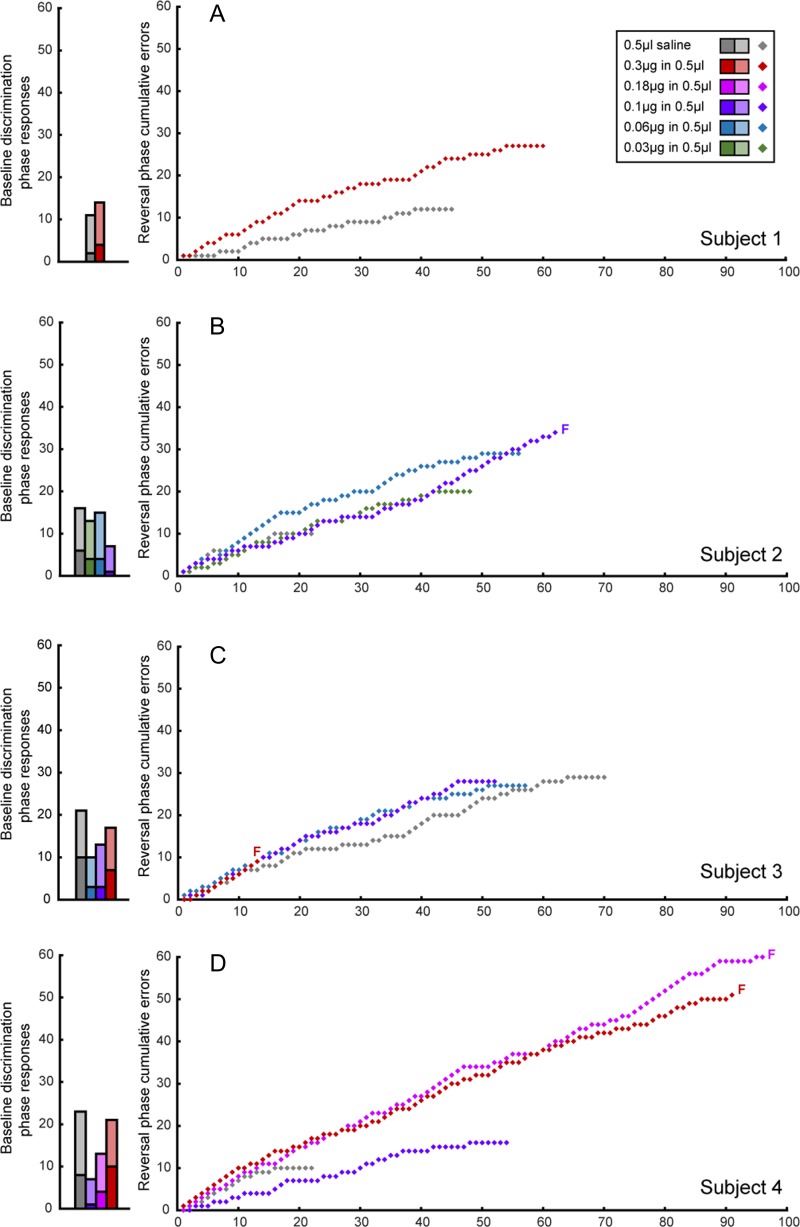
Effects of intra-putamen administration of muscimol and saline on the baseline discrimination and reversal phases of the task per subject. Baseline discrimination phase performance is displayed as bar graphs with the total number of responses split into correct (light shading) and incorrect trials/errors (dark shading). Reversal phase performance is shown as a learning curve, with cumulative errors plotted against trials. F denotes the failure of a subject to pass the relevant phase at a specific dose.

Individual trial-by-trial data are shown in Figure 4, which depicts cumulative errors over trials for each subject during the reversal phase. The gradient of the learning curve for intermediate intra-putamen doses of muscimol was steeper than that of the saline curve, indicative of a higher proportion of errors. There is little evidence of perseveration per se, as indicated by the lack of steep increases on the *y*-axis.

### Intra-Caudate Muscimol Infusions Produced Bi-Phasic Effects in Reversal Learning

Muscimol infusions into the caudate, similar to the putamen, also had graded dose effects dependent upon task phase. Whilst high doses impaired baseline discrimination and reversal, by contrast, low doses significantly improved reversal learning (Fig. 3B).

A mixed-model ANOVA on the error difference score confirmed a significant main effect of Dose (*F*(2,11.4) = 16.9; *P* < 0.001) and a strong trend toward a Phase by Dose interaction (*F*(2,10.5) = 3.60; *P* = 0.0643). While there was a significant effect of Dose in both baseline discrimination (*F*(2,7) = 8.95, *P* = 0.0118) and reversal phases (*F*(2,3.32) = 49.6, *P* = 0.00336), pairwise comparisons revealed a reduction in the error difference score specifically following low-dose intra-caudate muscimol as compared to saline (*P* < 0.0001) in the reversal phase. High-dose intra-caudate muscimol, in contrast, significantly increased errors both in the reversal (High vs. Saline: *P* < 0.00005, High vs. Low: *P* < 0.0001), and the baseline discrimination phase (High vs. Saline: *P* < 0.001, High vs. Low: *P* < 0.0005). Subject 4’s failed reversal data were excluded, because his impairment in baseline discrimination precluded his reaching reversal criterion in the allotted session time. There were no differences in performance across non-infusion sessions as measured by a raw error count (Supplementary Fig. S1B). A mixed-model ANOVA confirmed an effect of reversal (*F*(1,13) = 16.3, *P* = 0.0014), but no effect of Dose (*F*<1) or Phase × Dose interaction (*F*<1).

Similarly, intra-caudate muscimol doses significantly affected the trial count (*F*(2,13) = 14.8, *P* = 0.00044) and task phase (*F*(1,13) = 5.50, *P* = 0.036), with a trend level interaction between Phase and Dose (*F*(2,13) = 2.83, *P* = 0.096). Dose significantly influenced the number of trials performed in both baseline discrimination (*F*(2,7) = 11.7, *P* = 0.0059) and reversal (*F*(2,6) = 6.53, *P* = 0.031) phases. The reduction observed in reversal errors at low dose was reflected in the trial count at a trend level (Low vs. Saline: *P* = 0.053), while the high dose generally impaired performance compared to saline and low-dose muscimol during both the baseline discrimination (High vs. Saline: *P* < 0.00001; High vs. Low: *P* < 0.00001) and in reversal (High vs. Saline: *P* = 0.072; High vs. Low: *P* < 0.005), as it did with errors (Supplementary Fig. S2B).

Assessment of strategy usage to understand the low-dose muscimol reversal improvement (Supplementary Fig. S4) indicated that there was no effect of low-dose intra-caudate muscimol on win-stay as compared to the associated non-infusion control sessions (*F*<1). By contrast, there was a slight but non-significant increase in lose-shift responding (*F*(1,2) = 11.2, *P* = 0.079).

For the high-dose intra-caudate muscimol impairment, analysis of strategy use revealed a significant reduction in win-stay behavior (*F*(1,2) = 21.7, *P* = 0.043; Supplementary Fig. S5A) but no effect on lose-shift responding in the baseline discrimination phase (*F*<1). For the high-dose reversal impairment (Supplementary Fig. S5B), there was a similar but non-significant reduction in win-stay (*F*(1,2) = 2.45, *P* = 0.26).

**Figure 5.**
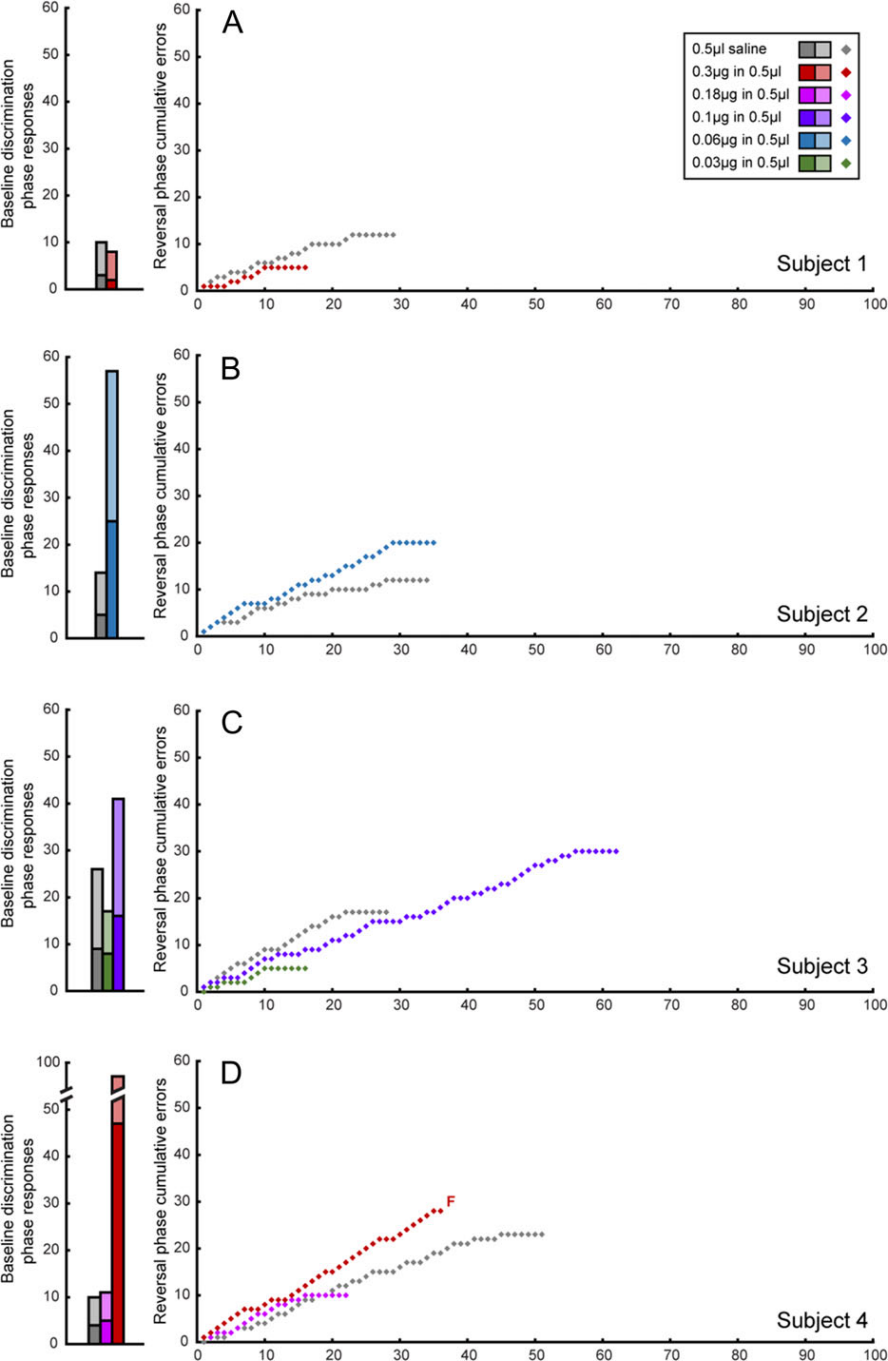
Effects of intra-caudate administration of muscimol and saline on the baseline discrimination and reversal phases of the task per subject. Baseline discrimination phase performance is displayed as bar graphs with the total number of responses in the baseline discrimination phase, split into correct (light shading) and incorrect trials/errors (dark shading). Reversal phase performance is shown as a learning curve, with cumulative errors plotted against trials. *F* denotes the failure of a subject to pass the relevant phase at a specific dose.

Individual learning curves are plotted for muscimol infusions into the caudate in Figure 5. In contrast to the steeper learning curves observed after intermediate dose intra-putamen infusions, shallower learning curves were evident following low doses of intra-caudate muscimol indicative of their improved performance.

### Assessment of Response Latencies Following Intra-striatal Muscimol Infusions

There were no effects (*P* > 0.1) of any factors (muscimol dose, task phase, or correct vs. error) on the latency difference score (median latency on day of infusion—median latency on preceding non-infusion day) in either putamen or caudate-associated datasets.

## Discussion

These results provide a new perspective on the relative roles of the putamen and caudate in reversal learning in primates. Intra-putamen and intra-caudate infusions of muscimol produced differential dose-dependent effects in marmosets performing a serial reversal learning task. Intermediate doses of muscimol into the putamen induced selective impairments in the reversal phase of the task, leaving performance unchanged in the baseline discrimination phase. At higher doses, subjects showed more profound, non-selective deficits that either increased errors across both phases of the task or caused the animals to disengage from the task during the reversal phase. In contrast, relatively low doses of intra-caudate muscimol improved reversal learning whilst a higher dose impaired baseline discrimination performance.

Three major features of these results deserve highlighting and provide important new insights into the differential roles of the putamen and caudate in visual discrimination learning and reversal. First, the anterior putamen, rather than the medial caudate, makes a positive contribution to serial reversal learning, as shown by the selective impairment in reversal following intermediate doses of intra-putamen muscimol. Second, the apparent opposing contribution of the medial caudate to serial reversal learning as shown by the improved rather than impaired performance in reversal following relatively low doses of muscimol into the medial caudate. Finally, third, the unexpected and pronounced deficits on baseline discrimination produced by higher doses of muscimol into the medial caudate, compared to the relatively inconsistent effects on this behavior of comparable doses of muscimol infused into the anterior putamen. In addition, it is also worth noting that there were marked individual differences in behavioral sensitivity to muscimol both between animals and between striatal regions within the same animal. The latter is perhaps not surprising given prior evidence of marked individual differences in the degree of GABA_A_ receptor binding in the striatum of rats which was shown to correlate with novelty- and amphetamine-induced locomotion (Gruen et al. 1996).

A key advantage of the present design of the serial discrimination reversal paradigm is the daily baseline discrimination phase, in which subjects are tested on the previous day’s response-outcome contingencies. The baseline phase of the task serves as a useful control during manipulations; if a subject can successfully discriminate between the stimuli in this baseline phase, any deficits seen in performance during the subsequent reversal phase can be assumed to be restricted to the special demands of the reversal itself, and not due to difficulties in other domains such as discriminative capability, attention, or motivation. Thus, these effects on other domains cannot easily account for the selective effects on reversal learning reported here following low to intermediate doses of muscimol infused into the putamen and caudate. Instead, it can be inferred that the putamen plays a selective facilitatory role and the medial caudate an antagonistic role, in flexible, adaptive responding during serial reversal performance. This conclusion is somewhat at odds with past findings both from our laboratory and others in which disruptions of caudate function in the marmoset (and dorsomedial striatum in rodents) impair reversal learning. However, it is broadly consistent with the evidence from (Groman et al. 2013) in vervet monkeys and in humans with focal lesions of the basal ganglia, which specifically affect the putamen (Bellebaum et al. 2008).

Deeper analysis of the reversal learning impairment after intra-putamen muscimol indicated that animals were less likely to stay with the newly rewarded stimulus immediately after reversal. This may be consistent with an account whereby the positive prediction error (win-stay) normally occurring following unexpected reward is blunted after putamen inactivation. However, the finding that a qualitatively similar (non-significant) reduction was apparent for lose-shift behavior suggests a more general loss of reinforcement sensitivity.

In explaining the putamen reversal deficit, it may also be necessary to take into account the over-learned nature of reversal learning in the present study. In previous marmoset and rodent studies, the animals have been relatively naïve to reversal learning, in marked contrast to the present study in which marmosets had received extensive training prior to cannulation. Such extensive training allowed for a stable baseline of serial reversal performance and thus facilitated the comparison of the effects of multiple doses of muscimol in the caudate and putamen across sessions. However, by achieving that stable baseline, animals’ responding may have undergone a shift from goal-directed actions to habits (Adams and Dickinson 1981a, 1981b; DeRusso et al. 2010; Smith and Graybiel 2013). Certainly, there is support in the literature for the formation of reversal learning sets over successive reversal problems involving not only novel discriminative pairs (Harlow 1949; Meyer 1951) but also the same pair of stimuli (Warren 1966; Gaffan and Harrison 1984; Gaffan 1985; Rygula et al. 2010; Jang et al. 2015). In particular, the development of learning sets has been demonstrated in marmoset monkeys across discrimination problems, reversal learning problems, and in serial reversal learning as described here (Cotterman et al. 1956; Miles and Meyer 1956; Rygula et al. 2010). That intra-putamen muscimol infusions impaired reversal learning performance is consistent with the proposal that this region of the striatum mediates habitual responding in rodents (Packard and Knowlton 2002; Graybiel 2008; Graybiel and Grafton 2015) and non-human primates (Fernandez-Ruiz et al. 2001; Miyachi et al. 1997, 2002; Deffains et al. 2010; but see Desmurget and Turner 2010).

Whilst habitual control over behavior has been considered primarily in the context of automatized stimulus-response associations (Dickinson 1985), we suggest that the application of well-learned rules of the form “if not A then B” (or “lose (A)—shift (to B)”) may become similarly automatized. We have shown previously that lesions of the ventrolateral prefrontal cortex, a key region in learning and generalizing rules in marmosets (Dias et al. 1996, 1997; Rygula et al. 2010) and humans (Bunge et al. 2003), only disrupt serial reversal learning when new stimuli are introduced, whereas lesions of the anterior OFC impair reversal learning regardless (Rygula et al. 2010). This suggests a different circuit is involved in applying the same rule, i.e., if not A then B, with novel visual stimuli, compared to familiar visual stimuli. We propose that the latter may be considered akin to an abstract, but still automatic, form of behavioral control, which, like simpler, more concrete stimulus-response associations, is dependent upon circuitry including the putamen. Indeed, an early study (Reading et al. 1991) showed that the acquisition of a visual instrumental discrimination task, which could be construed as an example of the application of a conditional rule to stimulus-response learning, was impaired by lesions of the lateral striatum (homologous to putamen) in rats. More recently, (Seymour et al. 2007) reported that activity in the putamen in a human fMRI study reflected negative prediction errors in reinforcement learning which may underpin the application of “A not B” (or “lose-shift”) rules.

The dependence on the putamen for successful serial reversal learning is in marked contrast to the apparent antagonistic role of the medial caudate nucleus, as reflected in improved reversal learning following low-dose intra-caudate muscimol. Unlike the putamen, the caudate has been implicated in flexible goal-directed behavior in rats (Kirkby 1969; Ragozzino et al. 2002; Castañé et al. 2010), monkeys (Divac et al. 1967; Clarke et al. 2008), and humans (Rogers et al. 2000; Cools et al. 2002), and a number of studies has shown the deleterious impact that the goal-directed and habit systems may have on one another if their balance is dysregulated. Such imbalances have been postulated and reported in a number of psychiatric disorders, including OCD and drug addiction (Gillan and Robbins 2014; Voon et al. 2015; Ersche et al. 2016). Thus, when a task is well-learned and controlled predominantly by the habit system, activation of the goal-directed system may become counter-productive, attempting to unnecessarily widen the search space for alternative responses following an error. Consequently, low-dose intra-caudate muscimol may facilitate control by the habit system through inhibition of the goal-directed system. This hypothesis is supported by the nearly significant increase in lose-shift responding that underpins the low-dose intra-caudate reversal improvement.

In contrast, the more general impairment in discrimination performance across both baseline and reversal phases produced by higher dose, as compared to lower dose, intra-caudate muscimol, may have arisen from a more potent or widespread inactivation. This generalized deficit is unlikely to have been due to impaired visual discrimination learning, since previously we have shown that lesions of the medial caudate nucleus, including the region targeted in the present study, did not impair the acquisition of a visual discrimination involving novel stimuli (Clarke et al. 2008). Such visual discrimination impairments have been associated with connections between visual association cortex and the tail of the caudate (Middleton and Strick 1996; Kim and Hikosaka 2015). Thus instead, the generalized deficit seen in the present study may have been due to other difficulties, such as identifying the currently rewarded stimulus in a context of high interference as a consequence of both stimuli having been rewarded on multiple previous occasions.

In terms of limitations of the present study, the order of infusions were not counterbalanced between sites, but it is difficult to envisage how this may have contributed to the pattern of results obtained. Certainly, baseline performance remained stable across the study. An additional limitation is on interpretation of the nature of the specific impact of different doses of muscimol on striatal output. At high enough doses the overall effect of muscimol will be to silence striatal output (e.g., Yin et al. 2006; Schilman et al. 2010; Bissonette and Roesch 2015). However, little is known about the relative sensitivity of, e.g., medium spiny output neurons versus various populations of interneurons within the striatum to GABA agonists. Thus, the paradoxical facilitatory effect of low-dose intra-caudate muscimol may therefore have arisen via release of medium spiny neurons from inhibition through GABA agonist-mediated hyperpolarization of inhibitory striatal interneurons. Alternatively, depolarization of striatal cholinergic interneurons (Login et al. 1998) may have affected performance through the release of acetylcholine onto muscarinic cholinergic receptors (Ragozzino et al. 2002). Finally, whilst infusions were targeted to sub-regions of the caudate and putamen that have previously been implicated in reversal learning, both in marmosets and other non-human primates, the possibility that other microcircuits within either area would be differentially affected cannot be ruled out.

In summary, the present study has provided causal evidence for a specific contribution of the putamen in serial reversal learning performance. Patients with neurological disorders such as Parkinson’s (Swainson et al. 2000) and Huntington’s diseases (Lange et al. 1995) or with focal lesions (Bellebaum et al. 2008) affecting the dorsal striatum, as well as neuropsychiatric disorders such as schizophrenia (Leeson et al. 2009) and OCD (Remijnse et al. 2005), have all been shown to exhibit simple visual reversal deficits under certain conditions, although it is less clear that the latter implicate the putamen specifically. The present report complements the growing literature highlighting the importance of the putamen in the control of well-learned stimulus-response habits, by extending its role to other, higher-order forms of automatic control whereby a simple conditional rule of the form “if not A, then B”, can be used to guide selection from a pool of over-trained stimulus–response associations (in this case, two). Such a role may for example, be important in explaining higher-order deficits in cognition in OCD patients recently shown to exhibit reduced functional connectivity between the dorsolateral prefrontal cortex and putamen in relation to cognitive performance (Vaghi et al. 2017).

## Supplementary Material

Supplementary Data
