# Supplementary material for: Selective Role of the Putamen in Serial Reversal Learning in the Marmoset
Source: Cereb Cortex. 2018 Nov 3;29(1):447–60. doi: 10.1093/cercor/bhy276 (PMC6294407; doi:10.1093/cercor/bhy276)
Supplement: Supplementary Data [file bhy276suppl_1.zip › bhy276_Jackson_CC-Suppl-subm2.docx]

**Supplementary Figure Captions**

**Supplementary Figure 1:** Error counts for non-infusion sessions associated with (A) putamen and (B) caudate. These data show performance stability across non-infusion sessions. The bars in each plot represent the mean for non-infusion sessions associated with saline and varying doses of intra-striatal muscimol (Sal=saline, Low=lower dose, Int=intermediate dose, High=higher dose, with subscripts “p” for “putamen” and “c” for “caudate”). There was a significant (p < 0.05) effect of reversal on error counts.

**Supplementary Figure 2:** Effects of intra-putamen (A) and intra-caudate (B) administration of muscimol and saline on the trial difference score (number of trials in the infusion session minus errors from the preceding control session) in the baseline discrimination and reversal phases of the task. The bars in each plot represent the mean for saline and varying doses of intra-striatal muscimol (Sal=saline, Low=lower dose, Int=intermediate dose, High=higher dose, with subscripts “p” for “putamen” and “c” for “caudate”). Data for individual subjects are denoted by the symbols defined in the tables below each plot, where specific doses are also tabulated. Faded grey text indicates that Subject 4’s high dose data was not included in the analysis of reversal performance (see Results). * = p<0.05 (# = p<0.1) in post-hoc tests.

**Supplementary Figure 3:** Assessment of response strategy following reversal in sessions impaired by intermediate doses of intra-putamen muscimol. Data were analyzed on a trial-by-trial basis to determine the probability of staying with the most recently selected stimulus after receipt of reward (left; “win-stay”) and shifting away from a stimulus following negative feedback (right; “lose-shift”). Proportions of win-stay and lose-shift were calculated for and compared across the day of infusion and preceding non-infusion session. The bars in the above plots represent the mean and symbols depict individual marmosets. * = p<0.05 (# = p<0.1) by rmANOVA.

**Supplementary Figure 4:** Assessment of response strategy following reversal in sessions improved by low doses of intra-caudate muscimol (see Supplementary Figure 3 for details). The bars in the above plots represent the mean and symbols depict individual marmosets. * = p<0.05 (# = p<0.1) by rmANOVA.

**Supplementary Figure 5:** Assessment of response strategy during baseline discrimination (A) and following reversal (B) in sessions improved by high doses of intra-caudate muscimol (see Supplementary Figure 3 for details). The bars in the above plots represent the mean and symbols depict individual marmosets. * = p<0.05 (# = p<0.1) by rmANOVA.
