# Supplementary figures and images for: Selective Role of the Putamen in Serial Reversal Learning in the Marmoset
Source: Cereb Cortex. 2018 Nov 3;29(1):447–60. doi: 10.1093/cercor/bhy276 (PMC6294407; doi:10.1093/cercor/bhy276)

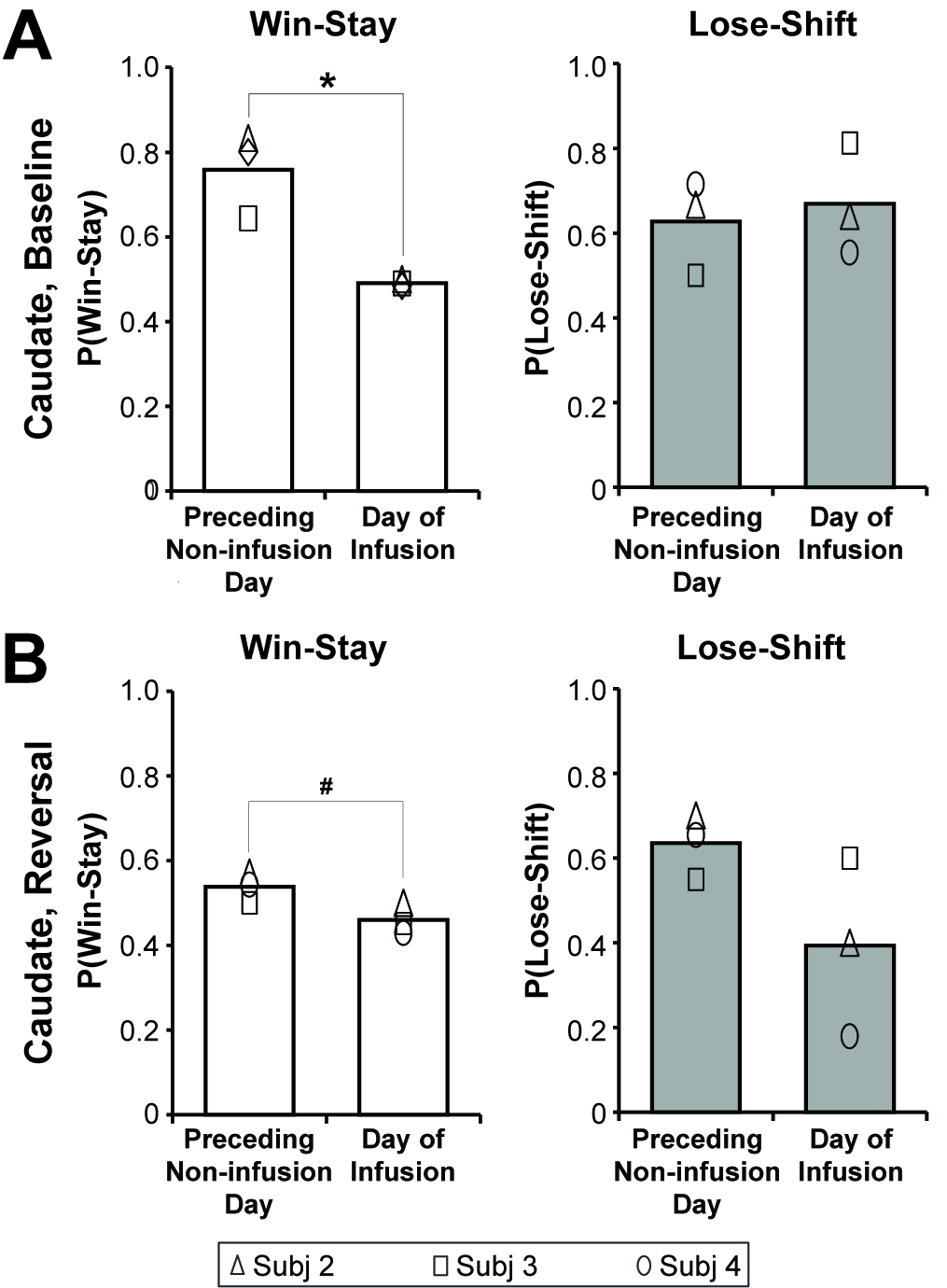

Supplement: Supplementary Data [file bhy276suppl_1.zip › bhy276_Supplementary_Figure_5.tif]

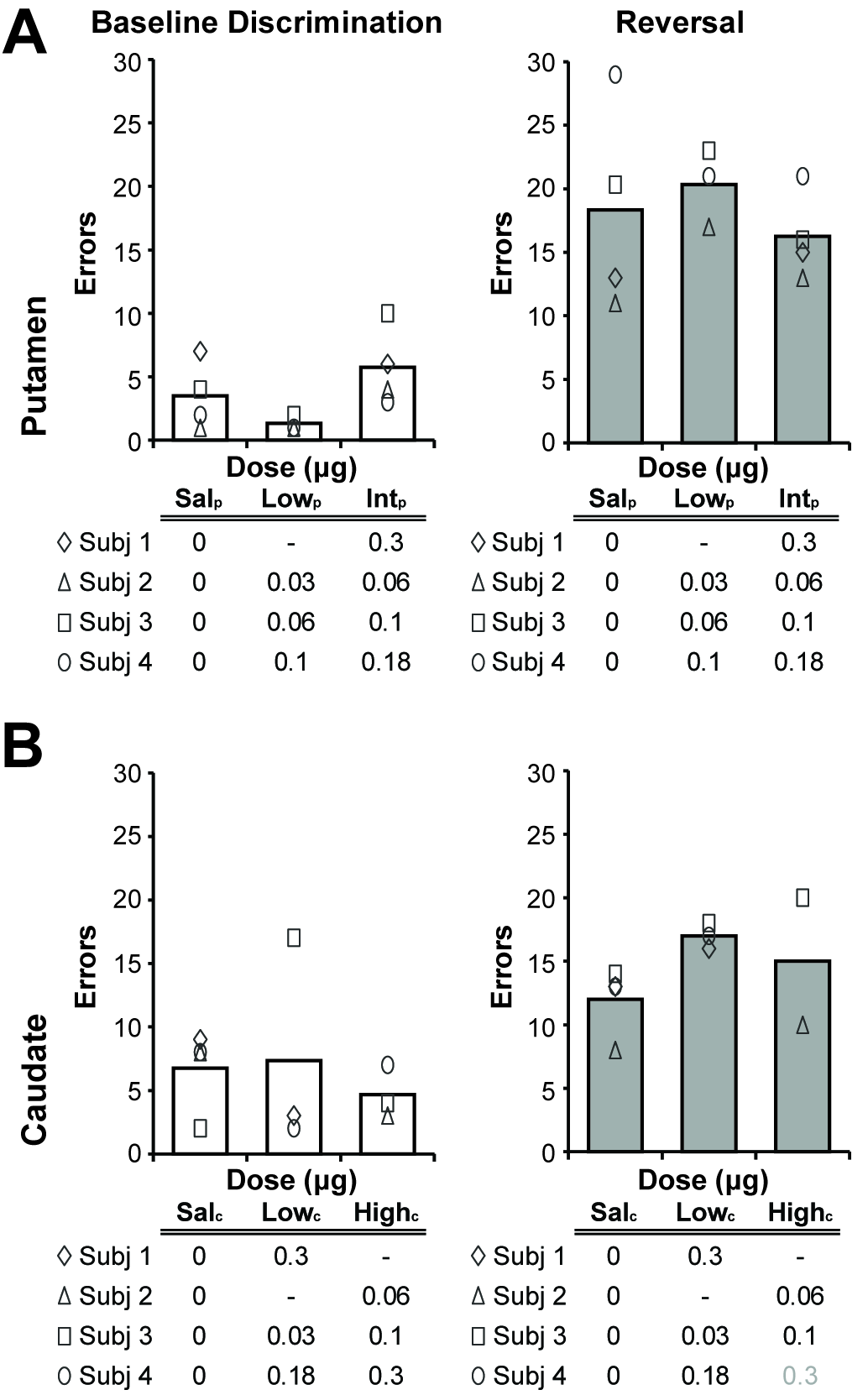

Supplement: Supplementary Data [file bhy276suppl_1.zip › bhy276_Supplementary_Figure_1.tif]

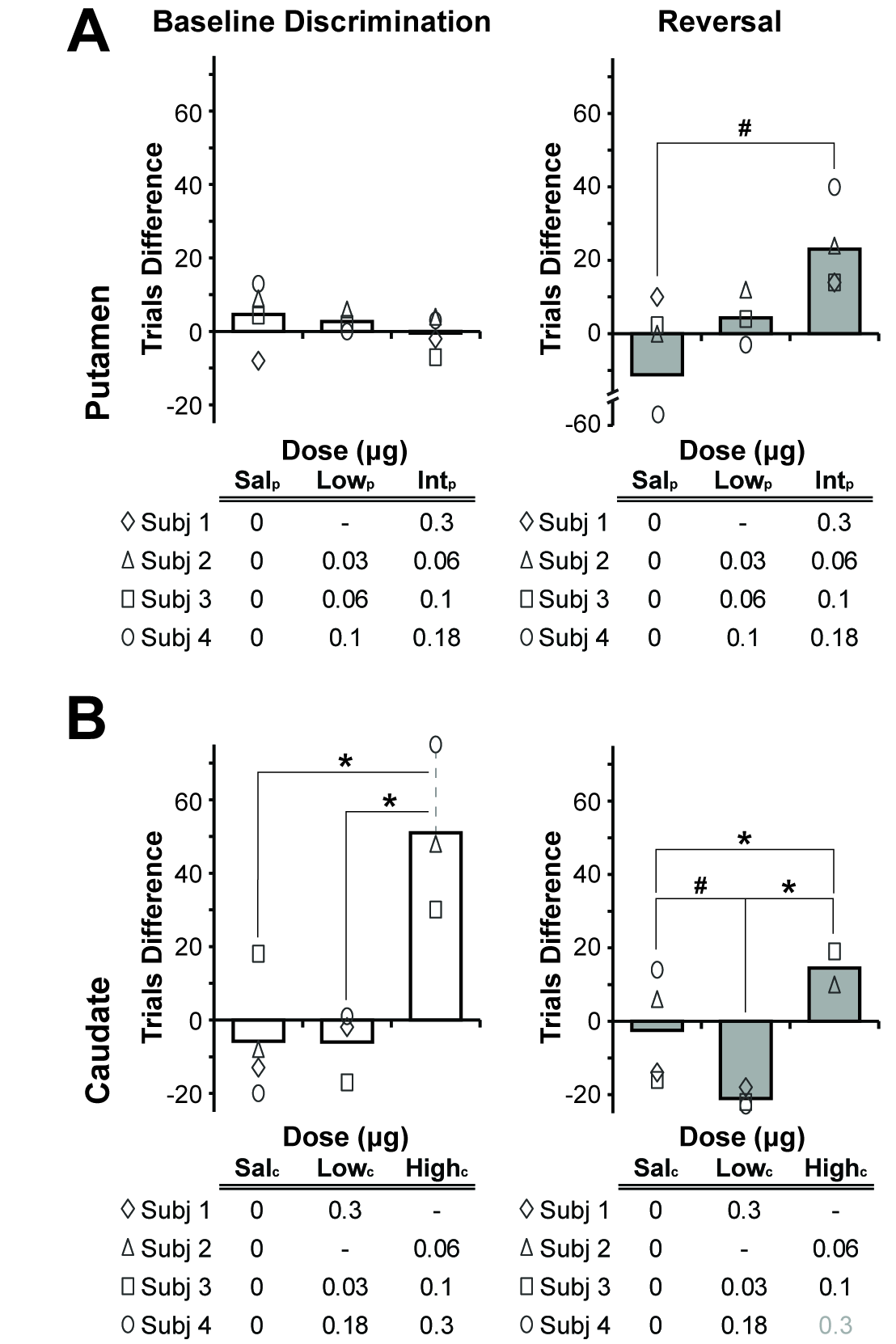

Supplement: Supplementary Data [file bhy276suppl_1.zip › bhy276_Supplementary_Figure_2.tif]

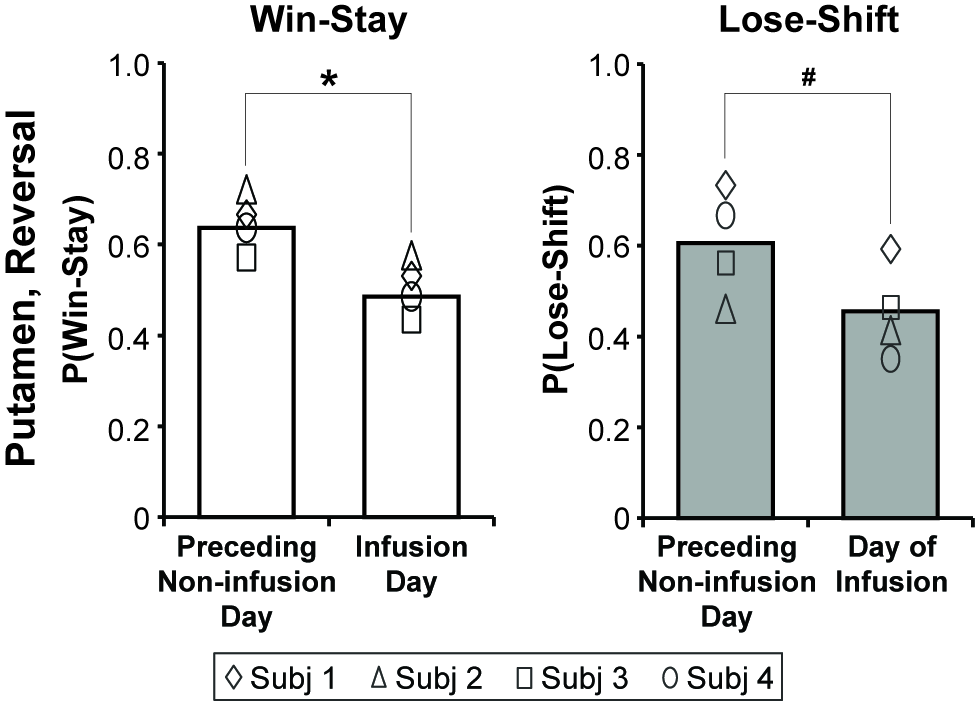

Supplement: Supplementary Data [file bhy276suppl_1.zip › bhy276_Supplementary_Figure_3.tif]

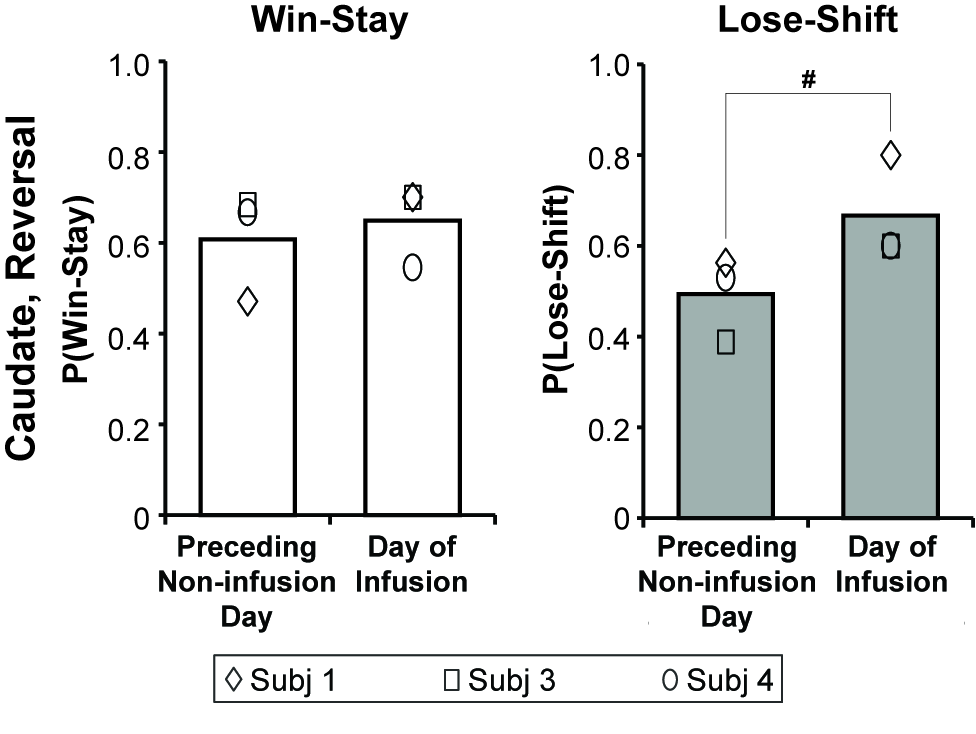

Supplement: Supplementary Data [file bhy276suppl_1.zip › bhy276_Supplementary_Figure_4.tif]
